# Atopy and Multisensitizations in Specific IgE Microarrays and Their Impact on Severe Asthma

**DOI:** 10.3390/life12101520

**Published:** 2022-09-29

**Authors:** Jan Romantowski, Aleksandra Górska, Grażyna Moszkowska, Julia Kulczycka, Karolina Minkowska, Agata Rolewicz, Marita Nittner-Marszalska, Marek Niedoszytko

**Affiliations:** 1 Department of Allergology, Medical University of Gdansk, 80-214 Gdansk, Polandmnied@gumed.edu.pl (M.N.); 2 Laboratory of Clinical Immunology and Transplantology, University Clinical Centre of Gdansk, 80-952 Gdansk, Poland; 3 Department of Medical Immunology, Medical University of Gdansk, 80-214 Gdansk, Poland; 4 Department of Internal Medicine Pneumology and Allergology, Wroclaw Medical University, 50-425 Wroclaw, Poland

**Keywords:** ALEX 1, microarray 2, IgE 3, asthma 4

## Abstract

(1) Asthma is a chronic inflammatory airway disease. Around 3–10% of patients experience severe refractory asthma. These patients with high symptom intensity and frequent exacerbations present a challenge for allergologists. Their allergic vs. non-allergic profile might be different from the standard asthmatic group and this difference is vital in qualifying for anti-IgE biologicals. The aim of the study was to analyze multiple sensitizations in patients with severe asthma and assess their impact on the course of the disease. (2) Forty-two patients with severe asthma according to GINA were enrolled. They experienced at least two exacerbations during the past year and had uncontrolled asthma despite high inhaled steroid use. A microarray serum Alex test (allergen-specific IgE to 295 extracts and components) was performed together with Complete Blood Count tests, the Asthma Control Questionnaire (ACQ), the Mini Asthma Quality of Life Questionnaire (MiniAQLQ), and spirometry. (3) There were 29 female and 13 male patients. The patient mean age was 50.4 (22–70). In 25 (60%) patients, inhalant sensitizations were detected. In 9 (21%) cases, a new perennial allergen was discovered that might enable anti-IgE treatment in the future. In the entire studied group, 8 patients (19%) would still not qualify for anti-IgE, anti-IL4, or anti-IL5 treatment. A linear regression analysis revealed that a *Canis familiaris* allergen (Can f 1) correlated with worse asthma control in ACQ. An *Aspergillus* allergen (Asp f 6) correlated negatively with Forced Expiratory Volume in one second (FEV1). (4) The study presents the usefulness of the ALEX test in 21% of patients with severe asthma in qualification for anti-IgE treatment. It highlights the impact of canine and *Aspergillus* sensitizations on worse control in patients with severe asthma.

## 1. Introduction

Asthma is a heterogenous chronic inflammatory lower-airway disease. Its symptoms (cough, wheezes, dyspnea, and chest tightness) change in intensity overtime, together with variable bronchi obstruction [1]. Due to modern inhalation drugs, namely Glucocorticosteroids (GCS) and long-acting beta-agonists (LABA), most patients achieve proper symptom control. However, approximately 3–10% of patients suffer from severe refractory asthma with very few additional therapeutic options [1,2]. These consist of recently developed biologicals: omalizumab, mepolizumab, benralizumab, reslizumab, dupilumab, and tezepelumab. Biologicals should be considered for every patient with severe asthma, namely those who do not achieve proper disease control despite using a high dose of inhaled GCS and LABA.

Although a selection of biological therapies is available, it must be noted that not all patients with severe refractory asthma qualify for such treatment [1,3,4]. Omalizumab is efficient only in patients with a significant inhalant allergy that is IgE-dependent. Thus, binding of serum IgE reduces the symptoms only in the case of allergic asthma. Mepolizumab, benralizumab, and reslizumab block either IL-5 or IL-5R, while dupilumab blocks IL-4. These drugs are efficient in asthma with high eosinophilic inflammation, reducing either exhaled nitric oxide or blood eosinophils. Finally, the recently introduced tezepelumab blocks TSLP so should reduce bronchial inflammation, regardless of the dominant mechanism [3,4]. This drug, however, is still not available in most countries.

In clinical practice, qualifying for biologicals consists of searching for either blood eosinophilia and/or inhalant sensitizations. This is mostly done via complete blood count tests (CBC), allergen-specific IgE detection in the serum using ELISA, or on the skin with skin prick tests. There is little option to improve the sensitivity of CBC testing beyond repeated assessments, though serum IgE detection might improve with the use of novel microarrays consisting of over 250 allergen extracts and molecules. Expanding the tested allergen profile makes it possible to find new sensitizations that are not detectable in a standard allergy work-up. Although the scientific reports are contradictory, in the allergologist community, allergic asthma is considered to respond better to standard inhalation treatment [4,5].

Common aeroallergens include house dust mites, cockroach antigens, molds, pets, and pollen [6]. Well-known triggers of allergic sensitization to fungi in humans are *Alternaria alternata*, *Aspergillus fumigatus*, and *Cladosporium herbarum* [7]. Fungi of the genus Alternaria and *Cladosporium* predominate in the external environment with their peak exposures from spring to fall. *Aspergillus* is a typical indoor mold species that favors development in conditions with high humidity and a lack of access to light and ventilation [7]. Fungal sensitivity is associated with severe asthma outcomes. However, the clinical implication of *Aspergillus fumigatus* sensitization in severe or difficult-to-treat asthma is still unclear and of interest to researchers [8].

As described above, there is a significant percentage of patients with severe refractory asthma who still cannot receive potentially life-saving biological therapy because of either a lack of inhalant allergy or high eosinophilia [1]. The prevalence of this group varies from 30% up to 50% of asthmatic patients. Detecting new sensitizations might help in the implementation of anti-IgE treatment.

Moreover, the patients might not be aware of uncommon inhalant sensitizations so uncovering new sIgE might help them to achieve proper control of the disease as allergen avoidance helps in reducing the number of asthma attacks [1,9].

The aim of the study was to assess the clinical relevance of the ALEX test in severe asthma qualification for anti-IgE treatment. The secondary aim was to analyze the correlation between molecular sensitizations and asthma severity in patients with severe asthma.

## 2. Materials and Methods

Forty-two adult patients with severe asthma according to GINA [1] were recruited (November 2020–December 2021). In line with inclusion criteria, all the patients had received a high dose of inhaled corticosteroids together with Long-Acting Beta-Agonists (LABA) and their asthma was still uncontrolled with at least two exacerbations within the past 12 months. Exclusion criteria were pregnancy, current anti-IgE treatment, and lack of consent. All the patients underwent a standard asthma and inhalant allergy workup, including skin prick tests, specific serum IgE, complete blood count tests with eosinophil count, Asthma Control Questionnaire (ACQ), Mini Asthma Quality of Life Questionnaire (MiniAQLQ), total daily prednisone dose, and spirometry [10,11,12,13]. The results of FEV1, VC, and FEV1/VC were presented in percentages of expected values according to Global Lung Initiative guidelines [13]. The daily prednisone dose was calculated to prednisone. Finally, in all the patients, the ALEX IgE microarray test was performed with 295 allergen components [14]. The total IgE concentration detection threshold was set at 20 U/mL and the positive cut-off point was set at 0.35 for sIgE. Statistical analysis consisted of non-parametric Spearman regression using Statistica 13 computer software. The analysis included correlation (linear regression) of all allergen sensitizations in the ALEX test with the ACQ, MiniAQLQ, total daily prednisone dose, Forced Expiratory Volume in one second (FEV1), Vital Capacity (VC), and FEV1/VC index. The spirometry outcomes were calculated according to the Global Lung Initiative. For comparisons between subgroups, the Mann–Whitney U test for independent values was used. In all tests, the cut-off point of the *p* value was set at 0.05. The study was approved by the Independent Boiethics Committee at the Medical University of Gdansk (approval number NKBBN/410/2019).

## 3. Results

**Figure 1:**
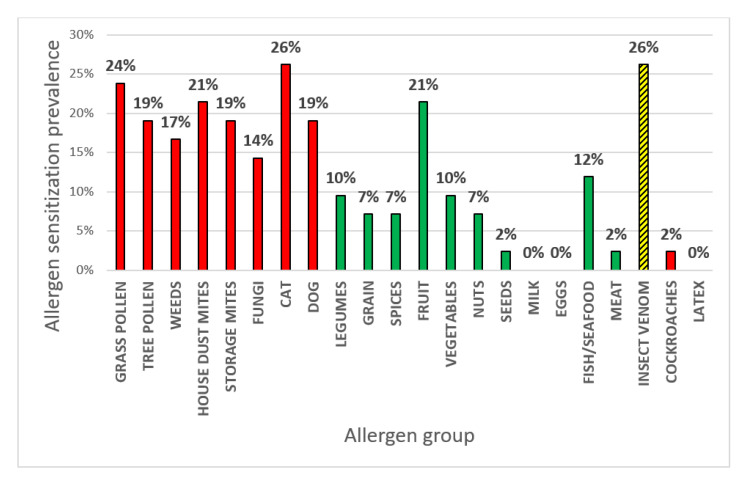
Prevalence of allergen (grouped) sensitizations according to ALEX test. Red indicates inhalant allergens, green—food, yellow/black—insect venom.

**Figure 2:**
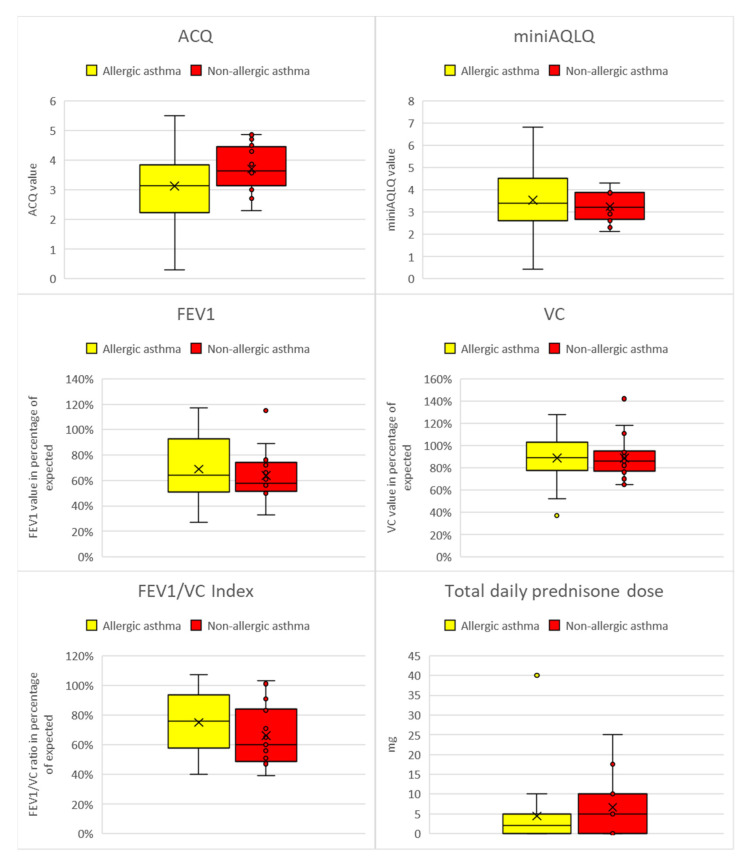
Comparison of allergic and non-allergic asthma. FEV1—Forced Expiratory Volume in 1 s; VC—Vital Capacity; ACQ—Asthma Control Questionnaire; MiniAQLQ—Mini Asthma Quality of Life Questionnaire.

There were 29 female and 13 male patients. The patient mean age was 50.4 (22–70). The results of the allergy and asthma work-ups are presented in Table 1, while the results of the ALEX tests with the allergen groups are presented in Figure 1. In 25 (60%) patients, inhalant sensitizations were detected. In this group, 9 (21%) subjects were considered to have non-allergic asthma in the standard allergy work-up, including SPT and serum IgE.8 patients (19%) who still would not qualify for anti-IgE, anti-IL4, or anti-IL5 treatment. Allergic asthma was considered if at least one inhalant sensitization was detected. No statistically significant differences were observed in the ACQ, MiniAQLQ, or spirometry values, or the total daily prednisone dose according to allergic or non-allergic asthma, though the results were slightly worse in non-allergic asthma (presented in Figure 2).

*Canis familiaris* allergen (Can f 1) correlated with worse asthma control in ACQ and lower Vital Capacity. *Aspergillus* allergen (Asp f 6) correlated negatively with FEV1. Both are considered to have a negative impact on severe asthma outcomes. Multiple allergens showed the opposite correlation (Bet v 2, Can f 3, Phl p 1, Phl p 2, Phl p 5, Phl p 6, Der p 7, Can f 3)—either decreasing ACQ or daily prednisone dose, or increasing MiniAQLQ or FEV1 and VC (Table 2 and Table 3). These would suggest a positive impact of a particular sensitization on asthma control.

## 4. Discussion

The prevalence of allergen sensitization in our study is similar to those found in the literature. The prevalence of allergen sensitization based only on skin test results in a multiethnic Asian cohort with severe asthma was as high as 78.2%, with sensitization to more than a single allergen in most cases [15]. Rojano et al. showed that patients with asthma are sensitized mostly to house dust mites (34%); however, pet sensitizations were also common (cat—33% and dog 21%) [16]. Kleine-Tebbe et al. also investigated patients with severe asthma and, in this group, similar allergens were found with more sensitizations in general (HDM—39%, cat—34%, dog—31%) [17]. This study also showed a high prevalence of grass pollen (*Phleum Pretense*) and birch pollen allergy (39% and 34%, respectively). In contrast, our study on severe asthma found that only 24% of patients were allergic to grass and 19% to trees, though a possible pattern is seen that, in the course of severe asthma, the most significant allergen may be pets. As presented in Figure 1, 14% of patients had fungi sensitizations which consisted mostly of *Aspergillus*. This is higher than the general population (9%) results and is in line with the results found in asthmatic patients (10–20% prevalence) [18,19].

Our study did not show a significant difference between allergic and nonallergic severe asthma. However, as seen in Figure 2, the allergic patients presented less severe results in all study outcomes without crossing statistical significance. Also, with the exception of Can f 1 and Asp f 1, most of the allergen sensitizations actually correlated with better asthma control. In the scientific literature, allergic asthma usually occurs at a younger age, is more frequently associated with rhinitis, and is more likely to affect men [20,21,22]. Nonallergic asthma is more likely associated with women and hypersensitivity to nonsteroid anti-inflammatory drugs (NSAIDs). The relation of asthma control and severity with IgE-related general sensitizations is still unclear in the scientific literature.

In our study, dog sensitization was found to be a factor that significantly worsens asthma control. Can f 1 is a lipocalin found in dog dander and might also correlate with lipocalins produced by cats (Fel d 7) [23]. Konradsen et al. showed that animal sensitizations to cats, dogs, and horses correlate with a severe asthma course [24]. A study by Konradsen et al. showed that patients with severe asthma sensitized to cats or dogs (28% in this group) have a worse response to omalizumab treatment both in disease control and FENO concentration [25]. In contrast, a study by Hom et al. showed that cat or dog sensitizations have no significant effect on atopic dermatitis, rhinitis, or asthma severity [26]. These results, together with our study, suggest that dog sensitizations may negatively influence asthma control, though only in patients who already have severe asthma.

As presented in Figure 1, 14% of patients had fungi sensitizations which consisted mostly of *Aspergillus*; other fungi such as *Aureobasidium*, *Alternaria* and *Cladosporium* that were found to be significant in other studies did not impact severe asthma in our study [27,28]. This is higher than the general population (9%) results and is in line with the results found in asthmatic patients (10–20%) [18,19]. A study by Rajagopal et al. showed that *Aspergillus* sensitization found in SPT worsens asthma control [29]. Our study shows that this effect results, in particular, from Asp f 6 (Table 2 and Table 3). Its negative influence on asthma is especially evident in the spirometry FEV1 values, though similar trends are observed in clinical asthma control outcomes without statistical significance. This molecule is a manganese superoxide dismutase (MnSod) and typically cross-reacts with other fungi allergens, i.e., Alternaria (Alt a 14) and Mallasezia (Mala a 14), and possibly with birch, olive, and ambrosia pollens [23,30,31]. *Aspergillus fumigatus* sensitization in difficult asthma identifies a more severe form of airway disease associated with greater morbidity, treatment need, and airways dysfunction/damage, but fewer psychophysiological comorbidities [8,32]. Importantly, severe asthma with fungal sensitivity (SAFS) is differentiated from allergic bronchopulmonary aspergillosis by the absence of bronchiectasis and fungal growth in the lungs and sensitivity to antifungal treatments [33]. The clinical course of asthma with fungal sensitization is similar to the clinical picture of asthma without sensitization to fungi except for the lower age of symptom onset and significantly higher levels of IgE and IL-33 in the serum [8,34,35]. However, studies focused on fungal sensitization in asthmatics are heterogeneous and have, to date, included differing asthma severities, making their conclusions difficult to interpret, particularly in the context of severe asthma [15]. Patients with *Aspergillus* sensitization had more exacerbations requiring steroid bursts, as well as evidence of airflow obstruction. No similar associations were found with sensitization to any other allergens [15]. 

Goh KY et al. showed a significant association between *Aspergillus* sensitization and uncontrolled asthma [15]. The prevalence of *Aspergillus*-specific sensitization was 11.7%. Moreover, patients with uncontrolled asthma had a higher rate of sensitization to *Aspergillus* (18.3% in uncontrolled asthma and 4.1% in controlled asthma, *p* = 0.001). No significant differences were found in the sensitization patterns to other allergens. Patients sensitized to *Aspergillus* were found to have higher steroid requirements (use of ≥2 steroid bursts in the past year), evidence of airflow obstruction, and one or more indicators of uncontrolled asthma compared with patients without *Aspergillus* sensitization.

Rajagopal et al. showed that the severity of asthma was associated with *Aspergillus* sensitization [29]. Uncontrolled symptoms were observed in only 28.07% of patients with asthma included in the study. The proportion of patients with uncontrolled symptoms was higher among patients who were sensitized to *Aspergillus* (45.00%) compared with patients who were not sensitized (18.92%). The proportion of patients with well-controlled symptoms was higher in individuals who were not sensitized compared with individuals who were sensitized (62.16% versus 50.00%, respectively).

Mistry et al. found A fumigatus sensitization in 23.9% of patients with difficult asthma [8]. Compared with A fumigatus non-sensitized subjects, those with sensitization were significantly more often male, older, with longer asthma duration, higher maintenance oral corticosteroid (39.7%) and asthma biologic use (27.6%), worse prebronchodilator airflow obstruction, and frequent radiological bronchiectasis (40%), but had less psychophysiological comorbidities.

Our study revealed 20% of Hymenoptera venom sensitizations. Due to geographical limitations, only bee and wasp IgE were found to be positive, and not that of ant venom. Though this result might seem high, it is still in line with other epidemiological studies. These estimate insect venom sensitizations at 23% of all people, while stinging is only symptomatic in 9% and might require treatment [36]. Interestingly, sensitizations to Hymenoptera and grass pollen often occur simultaneously so might be more frequent in allergic asthma populations [36].

An important finding was that in 21% of patients, new inhaled sensitizations were found, although these were considered non-allergic prior to the study. In this case, these patients might be able to receive omalizumab as a biological treatment according to GINA [1] though local regulators and payers might add other criteria such as whole-year allergen sensitizations [37].

Finally, the results point to the need of the implementation of new biologicals, since there is still a high number of patients (19%) who cannot receive the current biological therapy and who would most likely have to use high doses of standard therapy, oral GCS, which pose a risk of chronic side effects [1].

Potential study limitations were: (1) inclusion of only patients referred to by the Department of Allergology—patients not seeking specialist consultation for unknown reasons were not included; and (2) the study did not include patients who had already received anti-IgE treatment.

## 5. Conclusions

The study shows that the ALEX test can reveal new sensitizations in 21% of patients with severe asthma thus enabling anti-IgE treatment. Asp f 6 and Can f 1 correlate with a worse course of asthma in patients with severe refractory asthma.

## Figures and Tables

**Table 1:** Results of demography and asthma work-up. Expected values of pulmonary function tests were calculated according to Global Lung Initiative 2012 guidelines.

	Mean (Standard Deviation)
Age	50.4 (12.8)
ACQ	3.35 (1.14)
MiniAQLQ	3.41 (1.14)
FEV1 (of expected values)	66% (23%)
VC (of expected valued)	88% (20%)
FEV1/VC (of expected values)	71% (20%)
Daily prednisone dose	5.34 mg (7.7 mg)
Total eosinophil count	0.66 G/L (0.94 G/L)
Total number of patients	42
Average asthma duration in years	18.33 (11.24)
Number of inhaled sensitizations per patient	1.42 (n/a)
Number of patients with inhaled sensitizations pre study	15 (n/a)
Number of patients with inhaled sensitizations post study	24 (n/a)
Number of patients with detected new inhaled sensitizations	9 (n/a)

**Table 2:** Linear regression analysis of allergen sensitizations and asthma clinical outcomes: Asthma Control Questionnaire (ACQ) and Mini Asthma Quality of Life Questionnaire (MiniAQLQ). Red color indicates statistical significance.

	ACQ				MiniAQLQ			
Allergen	*p* Value	Beta	Confidence Interval Range		*p* Value	Beta	Confidence Interval Range	
Phl p 1	0.323	−0.158	−0.478	0.162	0.888	0.023	−0.301	0.346
Phl p 2	0.387	−0.139	−0.460	0.182	0.867	0.027	−0.297	0.351
Phl p 5	0.363	−0.146	−0.466	0.175	0.861	0.028	−0.295	0.352
Phl p 6	0.140	−0.235	−0.549	0.080	0.347	0.151	−0.169	0.471
Bet v 1	0.318	−0.160	−0.480	0.160	0.691	−0.064	−0.387	0.259
Bet v 2	0.026	−0.347	−0.650	−0.043	0.131	0.240	−0.075	0.554
Bet v 6	0.359	−0.147	−0.467	0.173	0.442	0.123	−0.198	0.445
Art v 1	0.286	−0.171	−0.490	0.149	0.643	0.075	−0.248	0.398
Art v 3	0.761	−0.049	−0.373	0.274	0.656	0.072	−0.251	0.395
Der f 1	0.560	−0.094	−0.416	0.229	0.936	−0.013	−0.337	0.311
Der f 2	0.342	−0.152	−0.472	0.168	0.890	0.022	−0.302	0.346
Der p 1	0.489	−0.111	−0.433	0.211	0.821	0.036	−0.287	0.360
Der p 2	0.335	−0.154	−0.474	0.166	0.874	0.026	−0.298	0.349
Der p 5	0.792	−0.043	−0.366	0.281	0.829	−0.035	−0.358	0.289
Der p 7	0.157	−0.225	−0.541	0.090	0.444	0.123	−0.199	0.444
Lep d 2	0.845	−0.031	−0.355	0.292	0.826	−0.035	−0.359	0.288
Tyr p 2	0.783	−0.044	−0.368	0.279	0.862	−0.028	−0.352	0.296
Alt a 1	0.056	0.301	−0.008	0.610	0.591	−0.086	−0.409	0.236
Alt a 6	0.755	0.050	−0.273	0.374	0.214	−0.198	−0.516	0.119
Asp f 1	0.470	0.116	−0.206	0.438	0.384	−0.140	−0.460	0.181
Asp f 3	0.397	−0.136	−0.457	0.185	0.537	0.099	−0.223	0.421
Asp f 4	0.253	−0.183	−0.501	0.136	0.534	0.100	−0.222	0.422
Asp f 6	0.458	0.119	−0.202	0.441	0.311	0.162	−0.158	0.482
Can f 1	0.014	0.381	0.082	0.681	0.060	−0.297	−0.606	0.013
Can f 2	0.761	−0.049	−0.373	0.274	0.656	0.072	−0.251	0.395
Can f 3	0.003	−0.457	−0.745	−0.169	0.013	0.383	0.084	0.682
Can f 4	0.840	−0.033	−0.356	0.291	0.847	−0.031	−0.355	0.293
Can f 6	0.761	−0.049	−0.373	0.274	0.939	−0.012	−0.336	0.312
Fel d 1	0.965	0.007	−0.317	0.331	0.372	−0.143	−0.464	0.177
Fel d 4	0.146	−0.231	−0.546	0.084	0.738	0.054	−0.270	0.377
Fel d 7	0.320	0.159	−0.161	0.479	0.218	−0.197	−0.514	0.121

**Table 3:** Linear regression analysis of allergen sensitizations and spirometry values: FEV1 and VC. Red color indicates statistical significance.

	FEV1				VC			
Allergen	*p* Value	Beta	Confidence Interval Range		*p* Value	Beta	Confidence Interval Range	
Phl p 1	0.018	0.367	0.066	0.668	0.076	0.280	−0.030	0.591
Phl p 2	0.004	0.441	0.150	0.732	0.043	0.317	0.010	0.624
Phl p 5	0.001	0.507	0.228	0.786	0.005	0.432	0.140	0.724
Phl p 6	0.005	0.429	0.136	0.721	0.020	0.362	0.060	0.664
Bet v 1	0.355	0.148	−0.172	0.469	0.435	0.125	−0.196	0.447
Bet v 2	0.243	0.186	−0.132	0.505	0.690	0.064	−0.259	0.387
Bet v 6	0.302	0.165	−0.154	0.485	0.986	−0.003	−0.327	0.321
Art v 1	0.127	0.242	−0.072	0.557	0.217	0.197	−0.121	0.514
Art v 3	0.875	−0.025	−0.349	0.299	0.467	0.117	−0.205	0.438
Der f 1	0.527	0.102	−0.220	0.424	0.339	0.153	−0.167	0.473
Der f 2	0.093	0.266	−0.046	0.578	0.334	0.155	−0.165	0.475
Der p 1	0.481	0.113	−0.209	0.435	0.294	0.168	−0.151	0.487
Der p 2	0.091	0.268	−0.044	0.580	0.318	0.160	−0.160	0.480
Der p 5	0.589	0.087	−0.236	0.410	0.433	0.126	−0.196	0.447
Der p 7	0.049	0.309	0.001	0.617	0.166	0.220	−0.095	0.536
Lep d 2	0.639	0.076	−0.247	0.398	0.480	0.113	−0.208	0.435
Tyr p 2	0.632	0.077	−0.246	0.400	0.482	0.113	−0.209	0.435
Alt a 1	0.951	0.010	−0.314	0.334	0.763	0.048	−0.275	0.372
Alt a 6	0.581	−0.089	−0.411	0.234	0.374	0.142	−0.178	0.463
Asp f 1	0.056	−0.301	−0.610	0.008	0.542	−0.098	−0.420	0.224
Asp f 3	0.748	0.052	−0.272	0.375	0.735	0.055	−0.269	0.378
Asp f 4	0.803	0.040	−0.284	0.364	0.750	0.051	−0.272	0.375
Asp f 6	0.037	−0.327	−0.633	−0.021	0.774	−0.046	−0.370	0.277
Can f 1	0.216	−0.198	−0.515	0.120	0.035	−0.330	−0.636	−0.025
Can f 2	0.875	−0.025	−0.349	0.299	0.467	0.117	−0.205	0.438
Can f 3	0.003	0.457	0.169	0.745	0.297	0.167	−0.152	0.486
Can f 4	0.616	0.081	−0.242	0.403	0.433	0.126	−0.196	0.447
Can f 6	0.847	0.031	−0.293	0.355	0.659	−0.071	−0.394	0.252
Fel d 1	0.540	−0.099	−0.421	0.224	0.234	−0.190	−0.508	0.128
Fel d 4	0.949	0.010	−0.314	0.334	0.738	−0.054	−0.377	0.270
Fel d 7	0.252	−0.183	−0.501	0.135	0.063	−0.293	−0.603	0.017

## Data Availability

Data will be available on request.

## References

[1] Global Initiative for Asthma (2022). Gina Main Report.

[2] Hekking P.-P.W., Wener R.R., Amelink M., Zwinderman A.H., Bouvy M.L., Bel E.H. (2015). The Prevalence of Severe Refractory Asthma. J. Allergy Clin. Immunol..

[3] Marone G., Spadaro G., Braile M., Poto R., Criscuolo G., Pahima H., Loffredo S., Levi-Schaffer F., Varricchi G. (2019). Tezepelumab: A Novel Biological Therapy for the Treatment of Severe Uncontrolled Asthma. Expert Opin. Investig. Drugs.

[4] Côté A., Godbout K., Boulet L.-P. (2020). The Management of Severe Asthma in 2020. Biochem. Pharmacol..

[5] del Giacco S.R., Bakirtas A., Bel E., Custovic A., Diamant Z., Hamelmann E., Heffler E., Kalayci Ö., Saglani S., Sergejeva S. (2017). Allergy in Severe Asthma. Allergy.

[6] Nelson H.S. (2000). The Importance of Allergens in the Development of Asthma and the Persistence of Symptoms. J. Allergy Clin. Immunol..

[7] Bozek A., Pyrkosz K. (2017). Immunotherapy of Mold Allergy: A Review. Hum. Vaccin. Immunother.

[8] Mistry H., Ajsivinac Soberanis H.M., Kyyaly M.A., Azim A., Barber C., Knight D., Newell C., Haitchi H.M., Wilkinson T., Howarth P. (2021). The Clinical Implications of Aspergillus Fumigatus Sensitization in Difficult-to-Treat Asthma Patients. J. Allergy Clin. Immunol. Pract..

[9] Rossi O.V., Kinnula V.L., Tienari J., Huhti E. (1993). Association of Severe Asthma Attacks with Weather, Pollen, and Air Pollutants. Thorax.

[10] Juniper E.F., Guyatt G.H., Cox F.M., Ferrie P.J., King D.R. (1999). Development and Validation of the Mini Asthma Quality of Life Questionnaire. Eur. Respir. J..

[11] Juniper E.F., Guyatt G.H., Epstein R.S., Ferrie P.J., Jaeschke R., Hiller T.K. (1992). Evaluation of Impairment of Health Related Quality of Life in Asthma: Development of a Questionnaire for Use in Clinical Trials. Thorax.

[12] Juniper E.F., O’Byrne P.M., Guyatt G.H., Ferrie P.J., King D.R. (1999). Development and Validation of a Questionnaire to Measure Asthma Control. Eur. Respir. J..

[13] Quanjer P.H., Stanojevic S., Cole T.J., Baur X., Hall G.L., Culver B.H., Enright P.L., Hankinson J.L., Ip M.S.M., Zheng J. (2012). Multi-Ethnic Reference Values for Spirometry for the 3-95-Yr Age Range: The Global Lung Function 2012 Equations. Eur. Respir. J..

[14] Heffler E., Puggioni F., Peveri S., Montagni M., Canonica G.W., Melioli G. (2018). Extended IgE Profile based on an Allergen Macroarray: A Novel Tool for Precision Medicine in Allergy Diagnosis. World Allergy Organ. J..

[15] Goh K.J., Yii A.C.A., Lapperre T.S., Chan A.K.W., Chew F.T., Chotirmall S.H., Koh M.S. (2017). Sensitization to Aspergillus Species Is Associated with Frequent Exacerbations in Severe Asthma. J. Asthma Allergy.

[16] Rojano B., West E., Ferdermann E., Markowitz S., Harrison D., Crowley L., Busse P., Federman A.D., Wisnivesky J.P. (2019). Allergen Sensitization and Asthma Outcomes among World Trade Center Rescue and Recovery Workers. Int. J. Environ. Res. Public Health.

[17] Kleine-Tebbe J., Mailänder C. (2020). Patterns of Allergen Sensitization in Patients with Severe Asthma in Germany. J. Allergy Clin. Immunol. Pract..

[18] Wps S.I. ECAP Epidemiologia Chorób Alergicznych w Polsce. www.ecap.pl..

[19] Hendrick D.J., Davies R.J., D’souza M.F., Pepys J. (1975). An Analysis of Skin Prick Test Reactions in 656 Asthmatic Patients. Thorax.

[20] Leynaert B., Sunyer J., Garcia-Esteban R., Svanes C., Jarvis D., Cerveri I., Dratva J., Gislason T., Heinrich J., Janson C. (2012). Gender Differences in Prevalence, Diagnosis and Incidence of Allergic and Non-Allergic Asthma: A Population-based Cohort. Thorax.

[21] Pakkasela J., Ilmarinen P., Honkamäki J., Tuomisto L.E., Andersén H., Piirilä P., Hisinger-Mölkänen H., Sovijärvi A., Backman H., Lundbäck B. (2020). Age-Specific Incidence of Allergic and Non-Allergic Asthma. BMC Pulm. Med..

[22] Takejima P., Agondi R.C., Rodrigues H., Aun M.V., Kalil J., Giavina-Bianchi P. (2017). Allergic and Nonallergic Asthma Have Distinct Phenotypic and Genotypic Features. Int. Arch. Allergy Immunol..

[23] Saarelainen S., Taivainen A., Rytkönen-Nissinen M., Auriola S., Immonen A., Mäntyjärvi R., Rautiainen J., Kinnunen T., Virtanen T. (2004). Assessment of Recombinant Dog Allergens Can f 1 and Can f 2 for the Diagnosis of Dog Allergy. Clin. Exp. Allergy.

[24] Konradsen J.R., Nordlund B., Onell A., Borres M.P., Grönlund H., Hedlin G. (2014). Severe Childhood Asthma and Allergy to Furry Animals: Refined Assessment Using Molecular-based Allergy Diagnostics. Pediatric Allergy Immunol..

[25] Sposato B., Scalese M., Milanese M., Masieri S., Cavaliere C., Latorre M., Scichilone N., Matucci A., Vultaggio A., Ricci A. (2018). Factors Reducing Omalizumab Response in Severe Asthma. Eur. J. Intern. Med..

[26] Hon K.L., Tsang K.Y.C., Pong N.H.H., Leung T.F. (2017). Relevance of Cat and Dog Sensitization by Skin Prick Testing in Childhood Eczema and Asthma. Curr. Pediatr. Rev..

[27] Niedoszytko M., Chełmińska M., Jassem E., Czestochowska E. (2007). Association between Sensitization to Aureobasidium Pullulans (Pullularia Sp) and Severity of Asthma. Ann. Allergy Asthma Immunol..

[28] Wagner G.E., Gutfreund S., Fauland K., Keller W., Valenta R., Zangger K. (2013). Backbone Resonance Assignment of Alt a 1, a Unique β-Barrel Protein and the Major Allergen of Alternaria Alternata. Biomol. NMR Assign..

[29] Rajagopal T.V., Kant S., Verma S.K., Kushwaha R.A.S., Kumar S., Garg R., Srivastava A., Bajaj D.K. (2020). Aspergillus Sensitization in Bronchial Asthma: A Separate Phenotype. Allergy Asthma Proc..

[30] Kleine-Tebbe J., Beyer K., Ebisawa M. (2016). Molecular Allergology User’s Guide. Pediatr. Allergy Immunol..

[31] Twaroch T.E., Curin M., Sterflinger K., Focke-Tejkl M., Swoboda I., Valenta R. (2016). Specific Antibodies for the Detection of Alternaria Allergens and the Identification of Cross-Reactive Antigens in Other Fungi. Int. Arch. Allergy Immunol..

[32] Twaroch T.E., Curin M., Valenta R., Swoboda I. (2015). Mold Allergens in Respiratory Allergy: From Structure to Therapy. Allergy Asthma. Immunol. Res..

[33] Denning D.W., O’Driscoll B.R., Hogaboam C.M., Bowyer P., Niven R.M. (2006). The Link between Fungi and Severe Asthma: A Summary of the Evidence. Eur. Respir. J..

[34] Masaki K., Fukunaga K., Matsusaka M., Kabata H., Tanosaki T., Mochimaru T., Kamatani T., Ohtsuka K., Baba R., Ueda S. (2017). Characteristics of Severe Asthma with Fungal Sensitization. Ann. Allergy Asthma Immunol..

[35] Zureik M., Neukirch C., Leynaert B., Liard R., Bousquet J., Neukirch F. (2002). Sensitisation to Airborne Moulds and Severity of Asthma: Cross Sectional Study from European Community Respiratory Health Survey. Br. Med. J..

[36] Nittner-Marszalska M., Liebhart J., Liebhart E., Dor A., Dobek R., Obojski A., Medrala W. (2004). Prevalence of Hymenoptera Venom Allergy and Its Immunological Markers Current in Adults in Poland. Med. Sci. Monit..

[37] Bartuzi Z., Bodzenta-Łukaszyk A., Kuna P., Kupryś-Lipińska I., Niżankowska-Mogilnicka E., Samoliński B. (2015). The Statement of the Polish Society of Allergology Regarding Necessary Changes in Therapeutic Program of Severe IgE-Mediated Allergic Asthma with Omalizumab. Adv. Respir. Med..

